# Minimally invasive direct lateral interbody fusion in the treatment of the thoracic and lumbar spinal tuberculosisMini-DLIF for the thoracic and lumbar spinal tuberculosis

**DOI:** 10.1186/s12891-018-2187-3

**Published:** 2018-08-07

**Authors:** Fengping Gan, Jianzhong Jiang, Zhaolin Xie, Shengbin Huang, Ying Li, Guoping Chen, Haitao Tan

**Affiliations:** Department of Orthopaedics, Guigang City People’s Hospital, No. 99-1 Zhongshan Rd, Guigang, 537100 People’s Republic of China

**Keywords:** Thoracic and lumbar spinal tuberculosis, Direct lateral interbody fusion (DLIF), Minimally invasive surgery

## Abstract

**Background:**

To investigate the clinical efficacy of minimally invasive direct lateral approach debridement, interbody bone grafting, and interbody fusion in the treatment of the thoracic and lumbar spinal tuberculosis.

**Methods:**

From January 2013 to January 2016, 35 cases with thoracic and lumbar spinal tuberculosis received direct lateral approach debridement, interbody bone grafting, and interbody fusion. Of the 35 cases, 16 patients were male and 19 were female and the median age was 55.2 (range 25–83). The affected segments were single interspace, and the involved vertebral bodies included: 15 cases of thoracic vertebrae (1 cases of T_5/6_, 2 cases of T_6/7_, 4 cases of T_7/8_, 3 cases of T_8/9_, 5 cases of T_9/10_) and 20 cases of lumbar spine (2 cases of L_1/2_, 6 cases of L_2/3_, 6 cases of L_3/4_, 6 cases of L_4/5_). After MIDLIF operation, all the patients received medication of four anti-tubercular drugs for 12 to18 months.

**Results:**

The patients were followed up for 7 to 40 months with an average of 18.5 months. The visual analogue scale (VAS) at the last follow-up was 2.8 ± 0.5, which was significantly different from the preoperative VAS (8.2 ± 0.7). After MIDLIF, there was 5 cases occurred with transient numbness in one side of the thigh or inguinal region, and 10 cases suffered from flexion hip weakness. All the bone grafts were fused within 6~ 18 months (average of 11.5 months) after the operation.

**Conclusion:**

Minimally invasive lateral approach interbody fusion technology have the advantage of less injury and quick recovery after surgery, which is the effective and safe treatment for thoracic and lumbar spinal tuberculosis.

## Background

In recent years, with the change of living environment, the epidemic situation of tuberculosis (TB) is still grim. China ranks second next to India among 22 high-burden countries despite decades’ effort on TB control [1]. The complications of Spinal tuberculosis including instability of the spine, spinal deformity and spinal cord compression, and even paralysis. Surgical therapy is recommended and required after the above complications occur [2]. However, traditional surgical treatment has the features of large trauma and slow postoperative recovery. Therefore, the development and application of various techniques in spinal surgery have provided new treatments for minimally invasive surgery for spinal tuberculosis. In our hospital from January 2013 to January 2016, there was 35 cases who treated with minimally invasive direct lateral approach tuberculosis debridement, bone grafting and internal fixation, and got a nice effect.

## Methods

### General information

A total of 35 cases (16 men and 19 women), whose age ranged from 25 to 83 years old (mean 55.2 years old), were investigated in this study. Clinical manifestations in the 35 patients included back pain, anorexia, fatigue, low-grade fever and sweats. There were 10 cases with spinal cord injury, and according to the Frankel grading [3], 4 cases of C class, and 6 cases of D class. The course of disease ranged from 3 months to 5.2 years, with an average of 13 months. The conventional digital X - line (DR), computed tomography (CT) and magnetic resonance imaging (MRI) examinations were performed before the operation, and then combination of the results of erythrocyte sedimentation rate (ESR) to produce a diagnosis as spinal tuberculosis. All the cases were confirmed by pathology. The affected segments were single interspace between adjacent vertebrae, and the involved vertebral bodies included, 15 cases of thoracic vertebrae (1 cases of T_5/6_, 2 cases of T_6/7_, 4 cases of T_7/8_, 3 cases of T_8/9_, 5 cases of T_9/10_) and 20 cases of lumbar spine (2 cases of L_1/2_, 6 cases of L_2/3_, 6 cases of L_3/4_, 6 cases of L_4/5_). Preoperative ESR was 46~ 110 mm/h, with an average of 71 mm/h. The preoperative mean progression of the Cobb angle was 25.2° (5°~ 48°) [4].

The inclusion criteria: ① patients with thoracic vertebrae tuberculosis from T5 to T11, and thoracolumbar tuberculosis from L1 to L5. ② patients who had necrotic bone, paravertebral abscess with segmental instability. ③ The lesions which were located in the anterior or middle regions of the spine.

Exclusion criteria: ①The lesions which were located in interspace between the L5 and S1 or above T5 thoracic, and thoracolumbar from T11 to L1. ② The lesions involvement of the posterior column (spinal accessory been affected), and projecting into spinal canal need posterior decompression. ③ open pulmonary tuberculosis. ④ multi-segmental or discontinuous spinal tuberculosis.

### Therapeutic method

#### Preoperative preparation

All the patients were treated with preoperative anti tuberculosis agents for more than 2 weeks before operation, including isoniazide (300 mg/d), rifampicin (450 mg/d), ethambutol (750 mg/d), streptomycin (750 mg/d) or pyrazinamide (15~ 30 mg/kg/day). During treatment, all the patients should receive periodic review of ESR, liver and kidney function, and the patient with the improvement of general condition (including mental, appetite etc.) would undergo surgery and were given glutamine to strengthen parenteral nutrition. The patients with the poor situation were given blood transfusions, Human Albumin etc. to support treatment.

#### Main instrument of experiment

Direct lateral interbody fusion (DLIF; Medtronic Sofamor Danek, Inc. Memphis, TN, USA) and Extreme lateral interbody fusion (XLIF; NuVasive, Inc., San Diego, California) side path minimally invasive fusion system are both used in side channel minimally invasive channel. Intraoperative neurological monitoring of patients was performed using the NIM-Eclipse (Medtronic, Medtronic Inc., Jacksonville, FL).

#### Operation method

After tracheal intubation general anesthesia, patients keeping 90 degree lateral position. The side that had bone destruction serious and more pus was chosen as surgical approach. DR marking surface location of vertebral disease, the lumbar bridge was aligned to the lesion segment of the vertebral body. Center on the waist bridge, the head and end of the operation table were both decreased about 40 degrees, and then properly fixed position. Preoperative C arm fluoroscopy determine and mark segmental lesions, and taking directly external incision of the abeam peritoneum in lumbar, about 5 cm, skin incision subcutaneous. In the C arm X-ray fluoroscopy guided, the blunt dissection of abdominal muscle layer (obliquus externus abdominis and obliquus internus abdominis) through the incision, the guide needle from the retroperitoneal space passed through the psoas muscle into the symptomatic vertebrae. The thoracic spine using the anterior lateral approach of the thoracic cavity, and taking a lateral oblique incision (about 6-8 cm) in midaxillary line which located on the upper interspace of the lesions interspace. Skin and subcutaneous incision, intercostal muscles and parietal pleura incision. Into the chest, separating the pleura. After lung protection with wet gauze, pushing forward the small S hook to show the lesion of intervertebral space. The guide wire was placed into vertebra clearance, and the C arm X-ray machine side perspective was used to determine the position of the positioning needle to ensure it was located in the middle position. Along the guide pin are inserted into the expansion sleeve and tubular separating hook, connecting to free arm and fixed. Proper opening of the lesion to reveal the intervertebral space, removal of paravertebral abscess and caseous material. Spatula, rongeur and chisel were used to remove lesions of intervertebral disc and necrosis of bone until health bone appearance. Rely on the spinous process of top pressure and intervertebral spreader correction of kyphosis, measuring bone groove length, implantation the same length autologous iliac bone grafting (18 cases), or select the appropriate length and diameter of titanium mesh (17 cases), in which 12 cases with the autogenous iliac bone implanted in titanium mesh bone graft and 5 cases with allogeneic freeze-dried bone cut into strips pieces implanted in titanium mesh bone graft, compaction and imbedding bone groove.

Internal fixation: 20 cases who showed no obvious osteoporosis and the stability of the spine is relatively small were treated with lateral anterior vertebral plate fixation, and 10 cases who showed serious damage of vertebral and intervertebral with posterior pedicle screw fixation,5 cases who did not showed serious damage but with obvious osteoporosis with lateral nail bar fixation. After irrigating the incision, streptomycin (1 g) and isoniazid (0.5 g) were placed in the gap. Thoracic vertebra and lumbar incision placement of closed thoracic drainage tube and drainage tube, respectively, and closed the incision. Intraoperative EMG monitoring was performed in all the 30 cases to avoid spinal cord and lumbosacral plexus injury.

#### Postoperative treatment

Postoperative routine treatment with antibiotics for 48 h, and thoracic cavity closed drainage tube was removed at 24~ 48 h after operation. During the postoperative 3–4 days, the patients were standing, walking and walking under the protection of a brace, and wearing a brace for 3 months. Continued anti-tuberculosis treatment for 12~ 18 months, regular review of liver, kidney function and ESR. The X-ray and CT examinations were performed at postoperative 1, 3, 6, 12 months and the last follow-up to evaluate bone fusion. The detailed surgical results of the 35 patients were presented in Table 1.

**Table 1:** The surgical results of 35 patients

No.	Gender	Age range	Segment lesion	Operation method	Follow-up time(month)	Pre-operation VAS score	Post-operation VAS score
1	male	26–30	L1/2	lateral anterior vertebral plate fixation	7	7	0
2	female	26–30	T7/8	posterior pedicle fixation	12	9	1
3	female	41–45	L2/3	lateral anterior vertebral plate fixation	18	8	1
4	female	61–65	T8/9	lateral anterior vertebral plate fixation	18	8	2
5	male	51–55	L4/5	lateral anterior vertebral plate fixation	24	9	1
6	male	46–50	T9/10	lateral nail bar fixation	21	8	0
7	female	76–80	T5/6	posterior pedicle fixation	15	8	2
8	female	66–70	L3/4	lateral anterior vertebral plate fixation	18	9	1
9	male	61–65	L4/5	lateral anterior vertebral plate fixation	22	7	0
10	male	56–60	T7/8	posterior pedicle fixation	20	6	2
11	female	46–50	L4/5	posterior pedicle fixation	36	8	1
12	male	31–35	T9/10	lateral anterior vertebral plate fixation	32	9	2
13	female	41–45	L1/2	lateral nail bar fixation	30	7	1
14	male	36–40	T7/8	lateral anterior vertebral plate fixation	24	7	1
15	male	76–80	L3/4	lateral nail bar fixation	22	8	3
16	female	81–85	T8/9	lateral anterior vertebral plate fixation	28	6	2
17	female	71–75	L3/4	lateral anterior vertebral plate fixation	24	7	0
18	female	61–65	T6/7	lateral anterior vertebral plate fixation	12	8	1
19	male	51–55	L4/5	lateral anterior vertebral plate fixation	18	7	0
20	female	56–60	T9/10	lateral nail bar fixation	18	9	1
21	male	46–50	L2/3	posterior pedicle fixation	24	7	1
22	male	31–35	L4/5	lateral anterior vertebral plate fixation	28	7	0
23	female	76–80	T6/7	lateral anterior vertebral plate fixation	36	8	2
24	female	71–75	L2/3	lateral anterior vertebral plate fixation	36	6	1
25	male	66–70	L3/4	posterior pedicle fixation	24	8	0
26	male	36–40	T8/9	lateral nail bar fixation	12	7	1
27	female	46–50	L4/5	lateral anterior vertebral plate fixation	28	8	1
28	female	56–60	L2/3	lateral anterior vertebral plate fixation	18	7	2
29	female	76–80	T7/8	posterior pedicle fixation	18	7	2
30	male	61–65	L3/4	posterior pedicle fixation	20	7	1
31	female	66–70	T9/10	lateral anterior vertebral plate fixation	24	6	1
32	female	66–70	L2/3	lateral anterior vertebral plate fixation	24	9	3
33	male	56–60	L3/4	posterior pedicle fixation	12	9	2
34	female	51–55	L2/3	posterior pedicle fixation	30	8	1
35	male	51–55	T9/10	lateral anterior vertebral plate fixation	12	7	2

*visual analogue scale: VAS

#### Declarations

This study obtained the written permission from all the participant.

## Results

The operation time ranged from 79 to 129 min, with an average of 94 min, and the perioperative blood loss was 200~ 600 ml, with an average of 320 ml. All cases were followed up for an average of 18.5 months (range from 7 to 40 months). After operation, the symptoms of back pain improved obviously, and the VAS score (2.3 ± 1.4) at the last follow-up was significantly lower than the preoperative score (8.2 ± 1.1). All wounds healed by first intention without any incision related complications, and no pulmonary infection, respiratory failure and other complications occurred. There was 5 cases occurred with transient numbness in one side of the thigh or inguinal region. Of the 5 cases, there was 4 cases, whose symptoms disappeared after 1~ 12 months of Neurotrophic therapy, and there was 1 cases, whose the symptoms of numbness were relieved after 12 months treatment, but did not disappear completely. There was 10 cases that suffered from flexion hip weakness and were recovered at 1~ 3 months without treatment. All patients had a good internal fixation at the last follow-up without complications such as loosening and breakage. The bone grafts were fused for 6~ 18 months, with an average of 11.5 months.

**Fig. 1:**
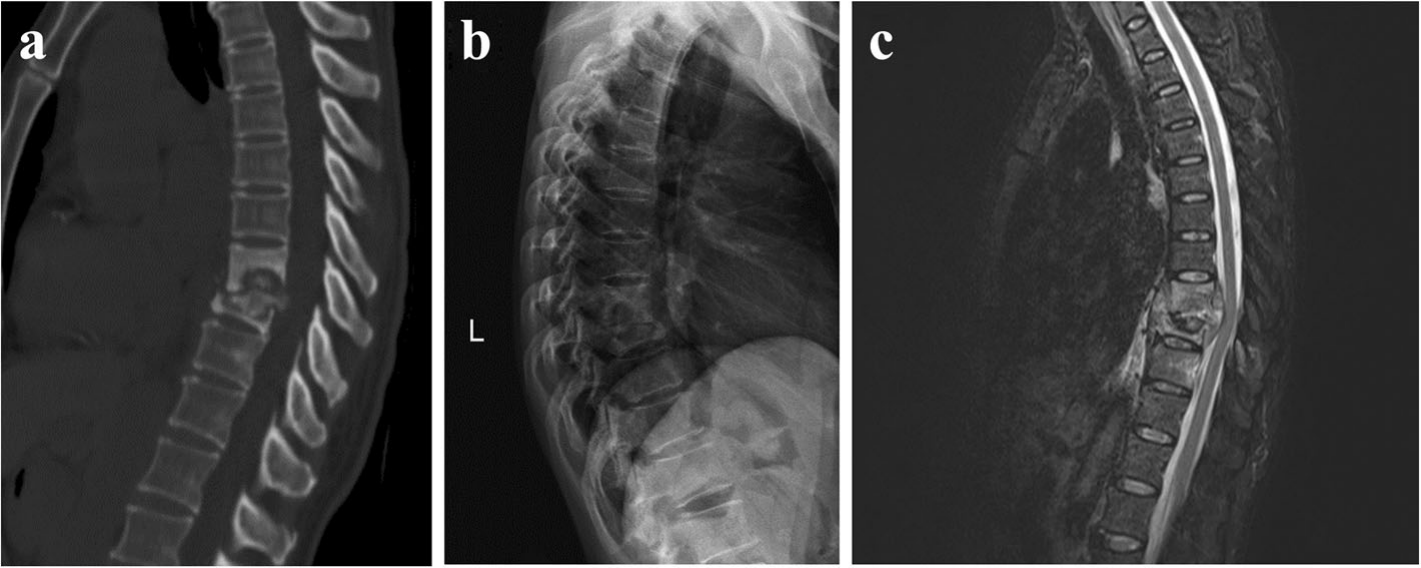
A patient with thoracic spinal tuberculosis at T7/8 received digital radiography (DR), computed tomography (CT) and magnetic resonance imaging (MRI) examinations before the operation. **a** Preoperative CT scan image showed bone defect at T7/8 with disc space narrowing. **b** Preoperative DR image. **c** Preoperative MRI image showed T7/8 vertebral tuberculosis with paraspinal abscess

**Fig. 2:**
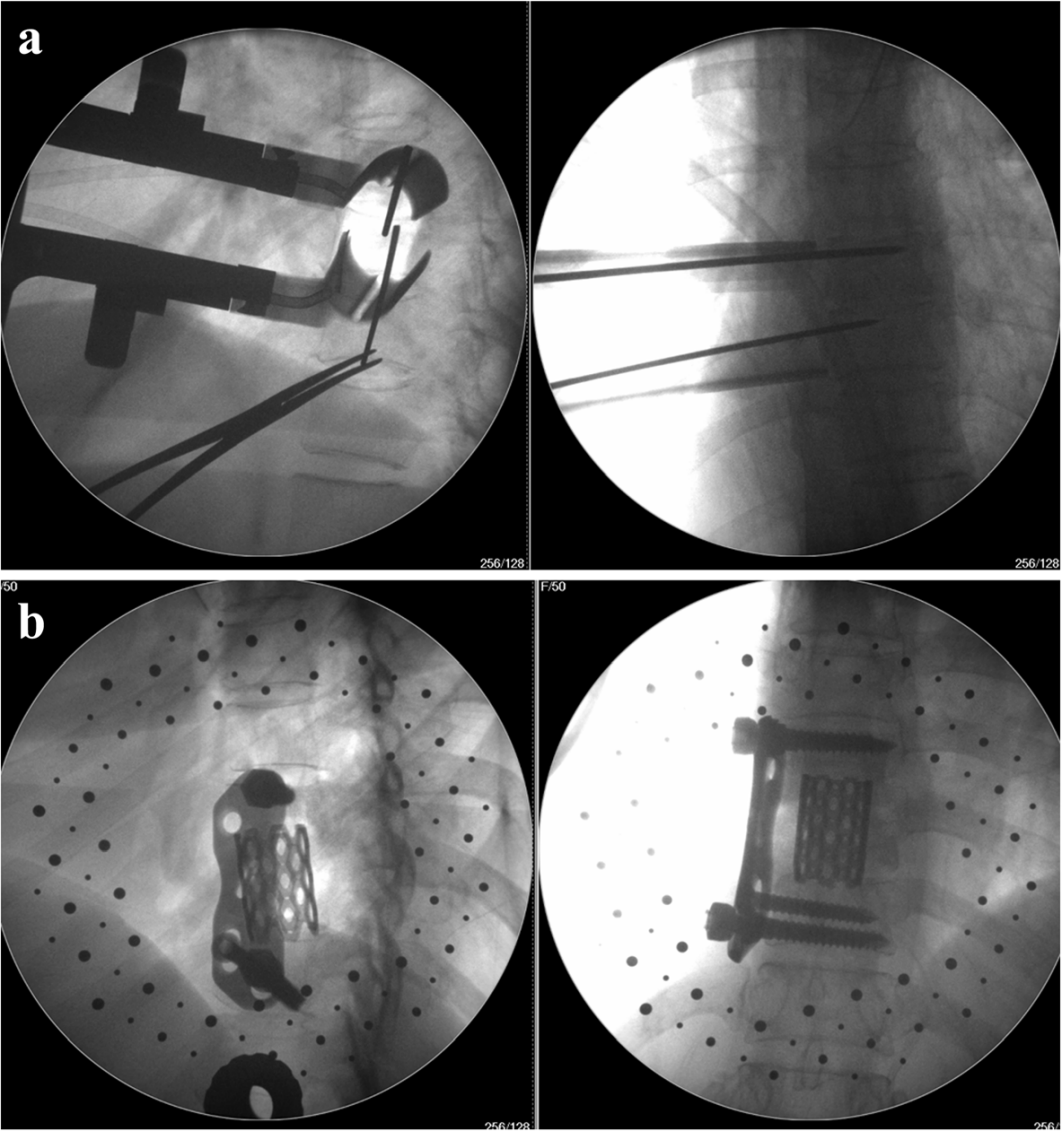
X-ray fluoroscopy of working channel in the operation. **a** The working channel established in the operation. **b** Postoperative X-ray after withdrawal the working channel

**Fig. 3:**
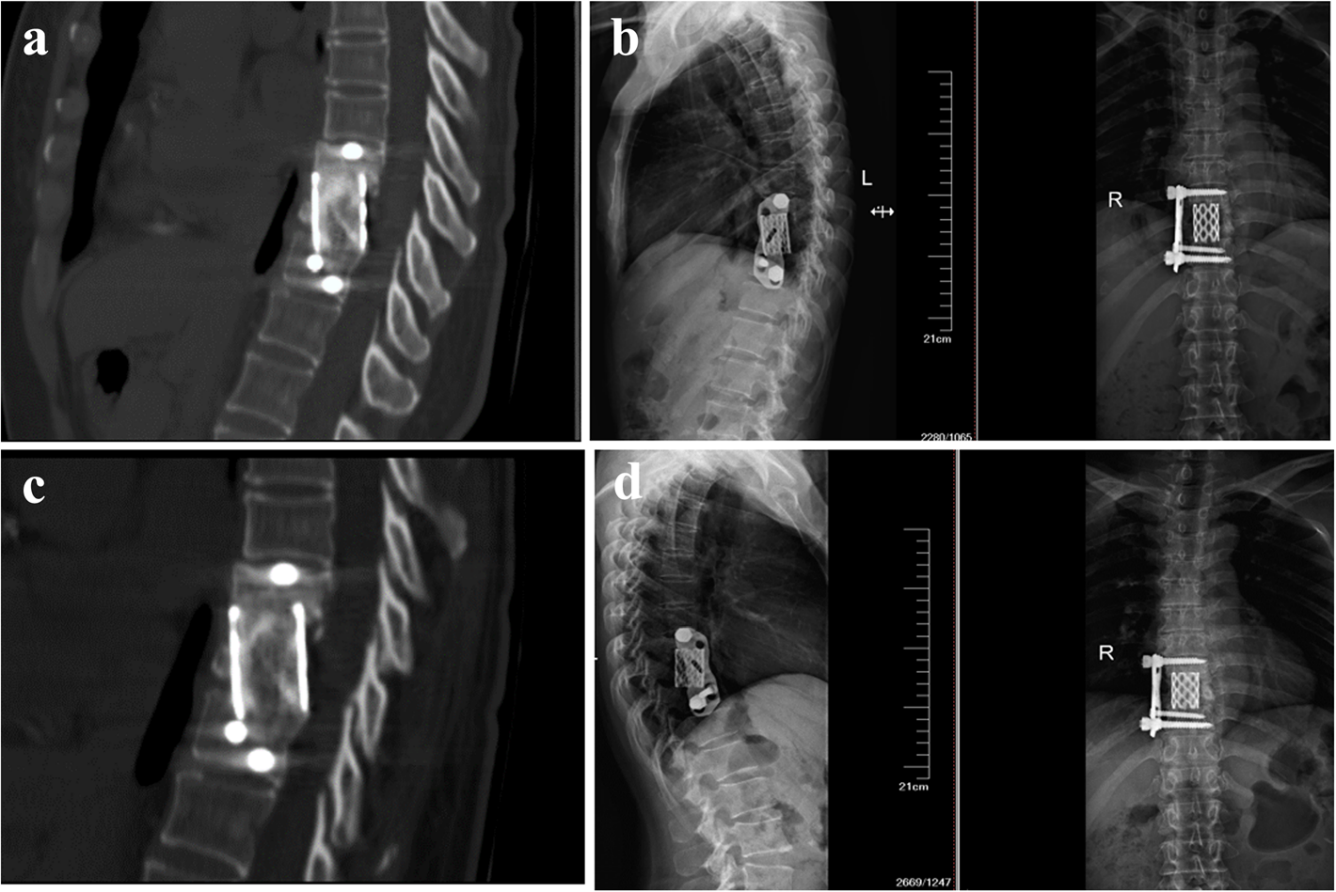
Postoperative DR and CT images showed good position of internal fixation and good bone fusion, respectively. **a** CT images of postoperative 18 months. **b** DR images of postoperative 18 months. **c** CT images of postoperative 24 months. **d** DR images of postoperative 24 months

As shown in Fig. 1, a 63 years old the female patient with thoracic spinal tuberculosis at T_7/8_ received digital X - line (DR), computed tomography (CT) and magnetic resonance imaging (MRI) examinations before the operation. Preoperative CT scan image showed bone defect at T_7/8_ with disc space narrowing and preoperative MRI image showed T_7/8_ vertebral tuberculosis with paraspinal abscess. The patient underwent minimally invasive direct lateral approach debridement, interbody bone grafting, and interbody fusion surgery (the autogenous iliac bone implanted in titanium mesh bone graft.), and Fig. 2 showed the working channel established in the operation and postoperative X-ray after withdrawal the working channel. The operation time was 86 min, and the perioperative blood loss was 550 ml (intraoperative blood loss 300 ml and postoperative blood loss 250 ml). After operation, the symptoms of back pain improved obviously, and the 1 week postoperative score (4.5) and 1 month postoperative score (1.8) were both significantly lower than the preoperative VAS score (8.9). Postoperatively 18 months and 24 months, the patient received DR and CT again, respectively, and the results indicated the good bone fusion and good position of internal fixation (Fig. 3).

## Discussion

Spinal tuberculosis (TB) is the most common form of extra-pulmonary tuberculosis, accounting for approximately 1 to 3% of all tuberculosis cases [5], and has a great impact on the quality of life, which can lead to paralysis. Spinal tuberculosis is local infection in systemic tuberculosis, the effective drug treatment is to kill *Mycobacterium tuberculosis*. Mild spinal TB respond well to the standard antituberculosis therapy, and early mild spinal tuberculosis with nutrition support and reasonable anti tuberculosis drugs and other conservative treatment can be cured [6].

The surgical treatment of spinal tuberculosis requires complete debridement, interbody fusion and internal fixation, and it can shorten the course of disease and reduce the fatality rate. But the traditional anterior spine fusion for the treatment of spine TB have large trauma, which will affect the treatment and rehabilitation of spinal tuberculosis [7–9]. Interventional therapy is a minimally invasive treatment method between conservative treatment and surgical treatment. Guided by CT and B-ultrasound, percutaneous micro-invasive treatment of local abscess and catheter drainage has the advantages of convenient operation, safe and effective, and less trauma, and is an effective and safe operation treatment for spinal tuberculosis abscess, especially be suitable for the general condition of patients [10]. But the postoperative problems were serious, such as the long indwelling drainage tube brings inconvenience to the life, the repeated blockage and the falling off of drainage tube, the retrograde infection and the long treatment cycle. In addition, the indications for interventional therapy are narrow and only applicable to the more abscess and which located in middle column of anterior spinal column. It is not suitable for the patients with instability, kyphosis, spinal cord compression, and the abscess locating in the spinal canal. Since the first thoracoscopic anterior spinal surgery performed by Mack in the early 1990s, thoracoscopic assisted anterior spinal surgery has developed rapidly. It has been developed from simple anterior discectomy and debridement to anterior spinal instrumentation and reconstruction, and has applications in a wide range of fields. A large number of literatures have confirmed its minimal invasion, safety and efficacy [11–13]. However, due to the higher requirements of surgical techniques, the need for more departments, longer learning curve, expensive equipment and other factors, there are also limitations of clinical application.

Direct lateral approach interbody fusion (DLIF) is a new technology in recent years. It used a minimally invasive approach single channel side could complete interbody fusion of single/multi segmental lumbar, restore intervertebral height, expand the nerve root canal, which achieved indirect decompression, alleviate root irritation symptoms, at the same time correction of scoliosis, vertebral tilt, slip deformity, rearrange and stabilize the spine. It was reported that DLIF was mainly applied in spinal degenerative diseases [14, 15]. Our hospital used DLIF in spinal degenerative disease which obtained good effect, and then it was applied in spinal tuberculosis. We have summarized some advantages of the minimally invasive direct lateral approach debridement, interbody bone grafting, and interbody fusion as following:① direct lateral small incision was about 5 ~ 6 cm, and only blunt separation of muscle fiber gap can reach directly to the affected vertebrae, which greatly reduced the incidence of other complications caused by surgical trauma. Since the direct access to the vertebral space which does not require destruction of any bony structure, the stability of the spine was better protected, and there was no risk of injury to important structures such as the large vessels and spinal cord without anterior or posterior approach. ②It can in the limited range of incision exposure reached complete debridement and bone graft and anterior lateral plate or vertebral pedicle screw fixation.

Our experience included:① strictly grasp the indications, mainly for T_4_ ~ T_11_ segment tuberculosis and L_2_~L_5_ segment tuberculosis, the lesions confined to the anterior and middle column and did not involve in the spinal canal and posterior column, and not need a posterior spinal canal decompression; ②accurate positioning of the preoperative and intraoperative opening baffle, it was arranged in the lateral 1/3 of the vertebral body to avoid injuring the lumbosacral plexus; ③ the most frequently reported complications of lumbar lateral extreme lateral interbody fusion (XLIF) was transient numbness in one side of the thigh or inguinal region and flexion hip weakness the with incidence rate of 1 ~ 60.1% [16]. In our study, there was 5 cases occurred with transient numbness in one side of the thigh or inguinal region, and the incidence rate was 14.3%. There was 10 cases suffered from flexion hip weakness, and the incidence was 28.6%. The main preventive measures are intraoperative electromyography (EMG) monitoring to avoid the injury of lumbosacral plexus during establishing passage. Most of the scholars emphasized that the application of EMG monitoring is very important to establish a safe approach [17]. EMG real-time monitoring can detect the distance between the expansion tube and nerve. The primary expansion tube inserted or opened every time was monitored by EMG, when the distance was 1 cm and nerve would send an alarm signal, the more closer and the more dense alarm. The signals by EMG guided the doctor to take measures to avoid the nerve, which greatly reduced the risk of lumbar plexus injury. Uribe et al. [18] reported that the application of electromyography can reduce nerve injury rate (less than 1%). There was 5 cases without intraoperative EMG monitoring, in which there was 2 cases occurred with transient numbness in one side of the thigh or inguinal region. The other 30 cases underwent real-time EMG monitoring in the working channel installation process. Once occurred abnormal evoked potential, the puncture direction immediately was changed, greatly reducing the risk of lumbosacral plexus injury in operation. However, there was one patient occurred with mild numbness in front of the thigh, the reason was EMG can only monitor the movement and cannot monitor the feeling, therefore it could not completely avoid nerve damage [19]. In addition, intraoperative EMG monitoring of XLIF may lead to false negatives, which required the doctor to pay a special attention to monitoring techniques during intraoperative EMG monitoring. Houten et al. [20] reported that there was 2 cases of lateral lumbar interbody fusion occurred postoperative motor deficits, but intraoperative electrophysiological monitoring showed no abnormalities. Our previous study have showed that, from August 2013 to October 2014, there was 46 cases with lumbar degenerative disease received XLIF in our hospital using real-time EMG monitoring, and there is nerve damage critical state or injury in 17 cases, including 3 cases of monitoring negative (false negative). Therefore, the surgeons are required to be familiar with the anatomical structures in the approach, observe the tissue similar to the nerve in the field carefully under the direct vision, operate carefully, and minimize the excessive traction of the psoas muscles and the compression of the surrounding soft tissue. The work channel was installed not too far back, as much as possible at the 1/3 junction of the vertebral body. Moreover, Rodgers et al. [21] reported that intravenous administration of 10 mg dexamethasone preoperatively could prevent the occurrence of nerve injury and significantly reduce the incidence of transient motor nerve injury.

## Conclusion

Direct lateral channel assisted approach can be used to perform resection and bone grafting and internal fixation, and it can be an effective and safe method for thoracolumbar tuberculotic spondylitis with the advantages of rapid recovery and less trauma.
